# Predictors of Breastfeeding Cessation among HIV Infected Mothers in Southern Ethiopia: A Survival Analysis

**DOI:** 10.1371/journal.pone.0090067

**Published:** 2014-03-07

**Authors:** Demewoz Haile, Tefera Belachew, Getenesh Birhanu, Tesfaye Setegn, Sibhatu Biadgilign

**Affiliations:** 1 Department of Public Health, College of Medicine and Health Sciences Madawalabu University, Bale Goba, Ethiopia; 2 Department of Population and Family Health, College of Public Health and Medical Sciences Jimma University, Jimma, Ethiopia; 3 Department of applied human nutrition, School of food sciences and Nutrition, Hawassa University, Ethiopia; 4 Department of Public Health, College of Medicine and Health Sciences, Bahir Dar University, Bahir Dar, Ethiopia; 5 Independent Public Health Consultants, Addis Ababa, Ethiopia; University of Cape Town, South Africa

## Abstract

**Background:**

Mother-to-child transmission (MTCT) of Human immunodeficiency virus (HIV) through breastfeeding remains the most significant route infection among children. Although the current guideline is recommending continued breastfeeding for HIV exposed infants, significant proportion of infants have been subjected to early weaning to prevent HIV transmission. However the predictors of breastfeeding cessation among HIV positive mothers were not documented in Ethiopia. Therefore the objective of this study was to determine the predictors of breastfeeding cessation among HIV-infected women in Southern Ethiopia.

**Methods:**

A facility based cross sectional study was conducted in Southern Ethiopia. The samples were selected by cluster sampling technique. The Kaplan-Meier curve was used to describe the survival time of breastfeeding and a step-wise multivariable Cox-proportional hazards regression model were used to identify the predictors of breastfeeding cessation. Both crude and adjusted hazard ratio were determined and p<0.05 was considered as statistically significant.

**Result:**

The mean duration of breastfeeding among HIV positive mothers was 13.79 [95% CI: (12.97–14.59)] months. The Kaplan-Meier estimate showed that proportions of women who were breastfeeding at 6, 9, 12 and 17 months were 89.3%, 75.3%, 66% and 17%, respectively. Those mothers having a monthly income of ≤500 ETB [AHR = 0.16, 95% CI :(0.03–0.76)], having a family size of three and below [AHR = 0.12, 95%CI: (0.02–0.68), four and above [AHR = 0.07, 95%CI: (0.01–0.35)] and bottle feeding [AHR = 3.95, 95%CI: (1.64–9.51)] were also independent factors associated with breastfeeding cessation.

**Conclusion:**

Above one third of HIV positive mothers stopped breastfeeding before 12 months. Monthly income, bottle feeding and family size were the independent predictors of breastfeeding cessations. Strengthening the current counseling and promotion modality on avoidance of bottle feeding and continued breastfeeding is recommended for improved HIV free survival.

## Introduction

Mother-to-child transmission (MTCT) of HIV has remained the most significant route of HIV infection among children [1]. In the absence of interventions during pregnancy and delivery, HIV transmission through breastfeeding could be responsible for over a third of all childhood HIV infections [2], [3]. Breast milk can be a source of HIV, but on the other hand, infants, particularly those in low-income settings, are at increased risk of malnutrition, diarrhea, acute respiratory infection, and death if they are not breastfed [4].

Multiple studies in low-income countries have documented increased morbidity and mortality associated with early cessation of breastfeeding compared to continued breastfeeding among HIV exposed children. Early cessation of breastfeeding to prevent HIV transmission would increase the risk of severe morbidities and mortality associated among HIV exposed infants who were weaned early (at 4–6 months of age), compared with those who breastfed (BF) for longer periods [5], [6], [7], [8].

A study from Zambia revealed that breastfeeding cessation is associated with malnutrition which showed a significant decrease in weight-for-age Z-scores among HIV exposed infants who stopped breastfeeding early (at 4 months) compared to those who breastfed continuously [9]. Although the risk of HIV transmission increased as breastfeeding continues, HIV free survival of HIV exposed infants who breastfed beyond 6 months was similar to those of infants who ceased breastfeeding at 6^th^ months [10], [11].

According to the revised national PMTCT guideline, HIV positive mothers are recommended to breastfeed exclusively for the first six months of life, followed by introduction of appropriate complementary feedings at sixth months with continued breastfeeding until 12–18 months [12]. However, there is a paucity of studies which show predictors of breastfeeding cessation among HIV positive mothers in Ethiopia. Identifying predictors of breastfeeding cessation among HIV infected women is important for targeting the education and counseling message to groups of women at risk for shorter breastfeeding which helps to increased HIV-free survival. The objective of this study is to assess the predictors of breastfeeding cessation among HIV positive mothers in Sidama Zone, Southern Ethiopia.

## Methods

### Ethics statement

Ethical approval was received from Hawassa University Institutional Review Board (IRB). Official letter of cooperation was also obtained from Sidama Zonal Health Department. The objective of the study was explained for the administrative body of the selected health institution and permission was obtained from the administration. The study objectives and procedures were explained and informed written consent was obtained from the respondents.

### Study setting and sample

In Sidama Zone, there were 18 health institutions which provide ART and PMTCT services. Four heath institutions were excluded because they had no eligible study subjects. Hence, the remaining eligible fourteen health institutions were considered as clusters. From the eligible 14 health institutions, ten health institutions (clusters) were selected randomly. A facility based cross sectional study was conducted in randomly selected 10 government health institutions (3 hospitals and 7 health centers) providing ART and PMTCT services in Sidama Zone Southern Ethiopia [13]. Considering the power of the study and sample adequacy, all (n = 184) HIV positive mothers who have HIV exposed infants aged 6–17 months found in the randomly selected health institutions were included.

### Data collection process and quality

Data were collected from all mother infant pairs found in the selected health institutions. The data were collected by health professionals recruited from respective health institutions and trained for two days on data collection techniques. The data collection process was closely supervised and the collected data were checked for completeness and consistency in the field.

Bottle feeding practices was measured by a 24-hour recall as recommended by WHO [14] and it was asked as “Did [Child Name] drink anything from a bottle with a nipple yesterday during the day or night time?”. Breastfeeding duration was assessed by asking the time lapse from date of birth to the time that a mother stopped breastfeeding. Those mothers who could not recall the month when they had stopped breastfeeding were excluded from the analysis.

### Statistical analysis

After the data collection was completed, data were checked for completeness and consistency; the data were entered, cleaned, and coded. Data analysis was carried out by SPSS for windows version 20 (IBM® SPSS® Statistics, IBM Corp, New York). Descriptive statistics were computed for all continuous and categorical variables. The Kaplan Meier (KM) curve with log rank test was used to describe the survival time of breastfeeding, probability of breastfeeding cessation and to compare survival curves by monthly income, family size and bottle feeding. Univariate and multivariable forward stepwise Cox-proportional hazards regression models were used. Those statistically significant variables (p<0.05) in univariate analysis were entered in to multivariable forward stepwise Cox proportional hazards regression model to identify the independent predictors of breastfeeding cessation. Both crude and adjusted hazard ratio (HR) with calculated 95% confidence interval were reported and p<0.05 was considered as statistically significant.

## Results

### Socio-demographic characteristics

The mean (+SD) age of mothers was 28.85 (+5.41) years while the mean (±SD) age of infants were 10.42(±3.49) months. Above thirty seven percent (37.5%) and 24.5% of the infants were in the age group of 6–8 months and 9–11 months respectively. Fifty seven percent (57%) of the infants were male by sex. Most of the mothers (77.7%) had an educational status of primary level and below. Majority of the mothers (85.9%) had disclosed their sero status at least to their husbands. More than two third (67.2%) of the mothers were on ART treatment while 32.8% of the mothers were on the pre-ART phase. One quarter of the infants had introduced complementary food before 6 months. Above eighty four (84.2%) and 64.7% of HIV positive mothers had ANC follow up and health institution delivery respectively (**Table 1**).

**Table 1:** Characteristics of HIV positive mother's infant pairs in Sidama Zone, Southern Ethiopia, 2012.

*Socio-demographic characteristics*	*Frequency*	*Percentage*
Age of the mother(years)		
≤24	28	15.6
25–29	73	40.8
≥30	78	43.6
Educational status		
Primary education and below	143	77.7
Secondary and above	41	22.3
Presence of animal milk at home		
Yes	33	82.1
No	151	17.9
ART status		
Pre ART	60	32.8
On ART	123	67.2
HIV sero status disclosure		
Yes	158	85.9
No	26	14.1
Current marital status		
Married	158	85.9
Not married	26	14.1
Place of residence		
Urban	134	72.8
Rural	50	27.2
Stigma and discrimination		
Yes	167	90.8
No	17	9.2
ANC attendance		
Yes	155	84.2
No	29	15.8
$Monthly income (n = 116)		
≤500	67	57.8
501–1000	29	25
≥1001	20	17.2
Family size(n = 182)		
≤3	50	27.5
4–5	103	56.6
≥6	29	15.9
Birth order(n = 176)		
1	54	30.7
2–3	94	53.4
≥4	28	15.9
When know sero status		
Before birth	156	84.8
After birth	28	15.2
Place of delivery		
Health institution	119	64.7
Home	65	35.3
Age of introduction complementary feeding		
Before 6 months	135	75.8
At 6 months and after	43	24.2
Age of infants (n = 184)		
6–8 months	69	37.5
9–11 months	45	24.5
12–17 months	70	38

$ - Exchange rate 1 USD = 17.87 Ethiopian Birr (ETB).

### Survival analysis for breastfeeding cessation

**Figure 1:**
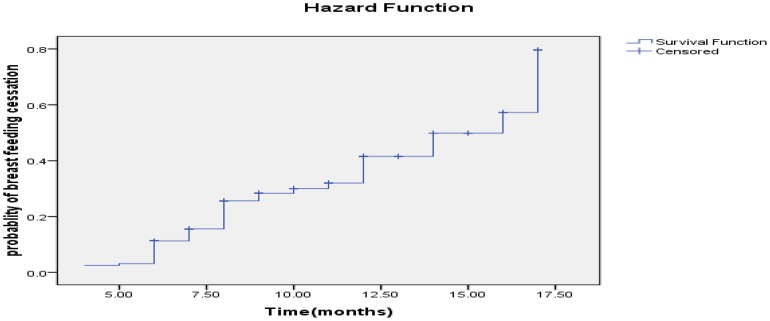
Probability of breastfeeding cessation of HIV positive mothers in Sidama Zone, Southern Ethiopia, 2012.

**Figure 2:**
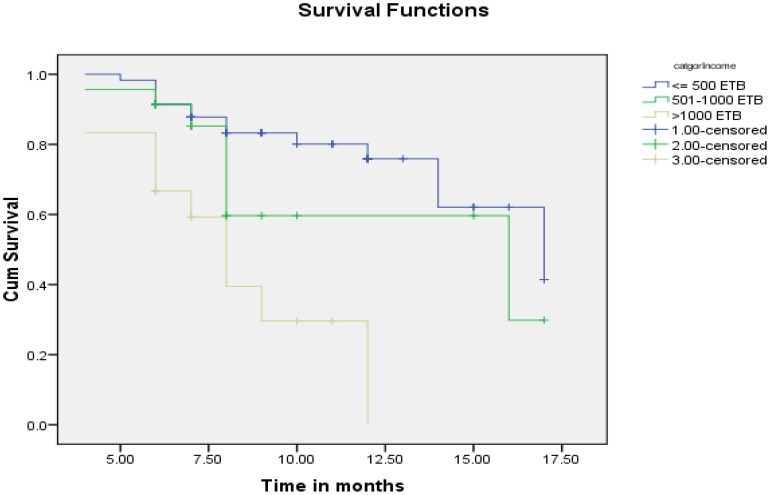
Survival function of breastfeeding duration stratified based on monthly income among HIV positive mothers in Sidama Zone, Southern Ethiopia, 2012.

**Figure 3:**
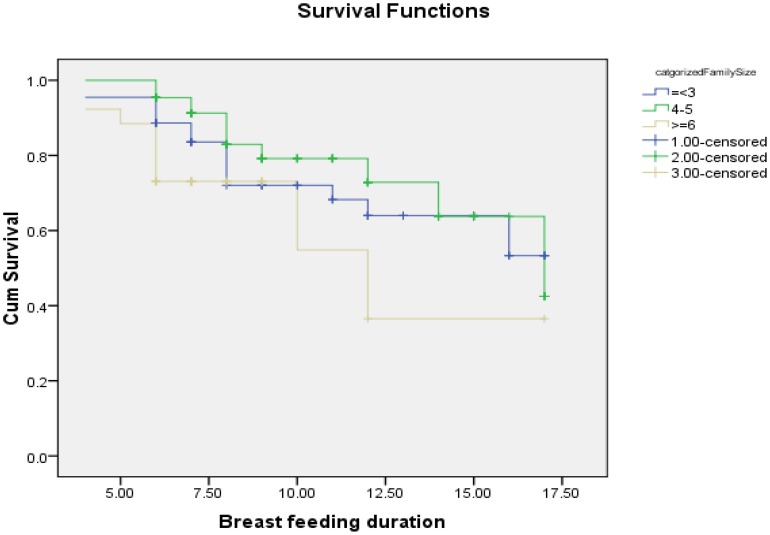
Survival function of breastfeeding duration stratified based on bottle feeding practices of HIV positive mothers in Sidama Zone, Southern Ethiopia, 2012.

The estimated KM proportions of women who were breastfeeding at 6, 9, 12 and 17 months were 89.3%, 75.3%, 66% and 17%, respectively (**Figure 1**). The mean duration of breastfeeding among HIV positive mothers was 13.79 [95% CI: (12.97–14.59) months. The median survival time for breastfeeding was 17 months. Twenty seven percent (27.7%) of HIV positive mothers had ceased breastfeeding at the time of the study. A test for comparing the equality of survival distribution between the different categories of income showed that there was a statistically significant difference in the survival distribution between different categories of income (Log Rank (Mantel-Cox)) test, chi squire = 19.266, Df = 2 p value = 0.001) (**Figure 2**). **Figure 3** **and** **4** shows the survival distribution of breastfeeding stratified based different categories of family size and bottle feeding respectively.

**Figure 4:**
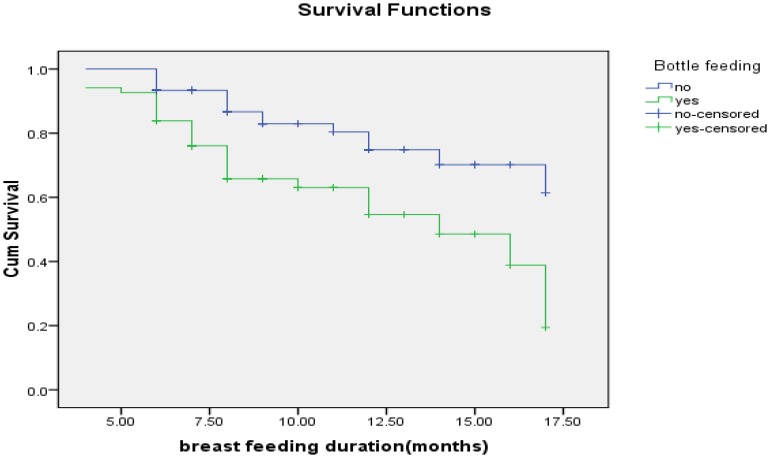
Survival function of breastfeeding duration stratified based on family size among HIV positive mothers in Sidama Zone, Southern Ethiopia, 2012.

The univariate analysis showed that mothers having animal(owned cows) milk at home, HIV status disclosure of the mother, educational status of mothers, monthly income, age of the mother, family size, infant age at initiation of complementary food, bottle feeding, place of residence and BMI(kg/m^2^) were significantly associated with cessation of breastfeeding (p<0.05).

Those HIV positive mothers having a monthly income of ≤500 ETB(Exchange rate 1 USD = 17 Ethiopian Birr (ETB)) [AHR = 0.16, 95% CI :(0.03–0.76)], having a family size of three and below [AHR = 0.12, 95%CI: (0.02–0.68), four and above [AHR = 0.07, 95%CI: (0.01–0.35)] and bottle feeding [AHR = 3.95, 95%CI: (1.64–9.51)] were also independent factors associated with breastfeeding cessation (**Table 2**).

**Table 2:** Univariate and multivariable analysis of the predictors of breastfeeding cessation among HIV positive mothers in Sidama Zone, Southern Ethiopia, 2012.

Variable	Crude HR [95%CI]	P-value	Adjusted HR[95%CI]	P value
Family size				
≤3	0.59[0.25–1.38]	0.22	0.12[0.02–0.68]	0.017
4–5	0.45[0.20–0.98]	0.047	0.07[0.01–0.35]	0.001
≥6	1			
Bottle feeding				
Yes	2.48[1.35–4.57]	0.004	3.95[1.64–9.51]	0.002
No	1		1	
Monthly income (ETB) *$				
≤500	0.19[0.09–0.45]	≤0.001	0.16[0.03–0.76]	0.021
501–1000	0.33[0.13–0.87]	0.025	0.93[0.29–3.03]	0.910
≥1001	1		1	
Age of the mother(years)				
≤24	0.37[0.14–0.98]	0.044	0.64[0.14–2.99]	0.575
25–29	0.39[0.19–0.79]	0.010	1.67[0.47–5.99]	0.433
≥30	1			
Presence of animal milk at home				
Yes	2.84[1.02–7.94]	0.04	3.87[0.41–36.47]	0.237
No	1		1	
HIV status disclosed				
Yes	1			
No	0.19[0.046–0.79]	0.02		
Educational status of mothers				
Primary and bellow	2.16[1.02–4.57]	0.04		
Secondary and above	1			
Age at introduction of complementary food				
Before 6 months	1.99[1.03–3.85]	0.04	0.86[0.23–3.23]	0.822
At 6 months and after	1			
Place of residence				
Urban	2.15[0.91–5.08]	0.08		
Rural	1			
BMI((kg m−2)				
<18.5	0.44[0.17–1.11]	0.08		
18.5–24.9	0.39[0.17–1.08]	0.05		
≥25	1			
Getting any food or cash support				
Yes	1			
No	0.92[0.46–1.83]	0.82		
ART status				
Pre ART	0.69[0.35–1.37]	0.28		
On ART	1			
Current marital status				
Married	0.99[0.47–2.11]	0.99		
Not married	1			
When you know your sero status				
Before birth	0.20[0.72–5.66]	0.18		
After birth	1			

## Discussion

Optimizing HIV free survival for infants born from HIV infected mothers is a major challenge in sub-Saharan Africa, where prevalence of HIV infection remains high among reproductive age women. The current WHO guideline emphasizes the importance of continued breastfeeding to improve HIV free survival in resource limited areas [5].

In this study, 66% of HIV positive mothers were breastfed their HIV exposed infants for 12 months. This is very low as compared to the study from Tanzania which report that 95% of HIV positive mothers breast fed at 12 months [15]. But a study from Brazil revealed that only 33.7% of HIV positive mothers breast fed their infants at 12 months which is lower as compared to our finding [16]. The mean breastfeeding duration of this study was shorter as compared to the study done in Tanzania [15].

Breastfeeding cessation was associated with presence of animal milk at home (milk from owned cows). This might be due to the fact that mothers who have animal milk at home provide cow milk as a replacement of breast milk and stop breastfeeding earlier due to the fear of HIV transmission through breastfeeding. Similarly, monthly income was also independent predictor of early breastfeeding cessation. This might be due to the fact that those mothers who have relatively better monthly income would have the capacity to afford complementary food. Similar studies from South Africa and Uganda have revealed that mothers who were economically better-off were more likely to introduce complementary food for their infants at an early age compared with economically poor mothers [17], [18], [19]. In this study, having larger family size is protective of early breastfeeding cessation. This might be related to the economic capacity of the household to afford other foods to replace breast milk to prevent mother to child transmission of the virus through breastfeeding.

This study found that introduction of food before 6 months was not a predictor of breastfeeding cessation in the multivariable model. However other studies in both developing and developed countries which showed introduction of cow's/powdered milk and infant formula were associated with earlier cessation of breastfeeding [15], [20], [21], [22].

Our study did not show statistically significant association between mothers' educational status with early cessation of breastfeeding. But disclosure of HIV status was statistically associated with breastfeeding cessation. In these regards a study done in Kisumu showed that the educated mothers are more likely to stopped breastfeeding [23].

The study from Tanzania reported that instrumental and material support (which included help with domestic chores, monetary assistance, transportation, etc.) was associated with delayed cessation of breastfeeding [15]. However this study did not find any statistically significant association between getting any food or cash support and cessation of breastfeeding.

### Limitations

There are two major limitations in this study. The study design was institutional based cross sectional study so that the breastfeeding duration was determined by asking the mother to recall the time when she had stopped breastfeeding and this study did include HIV exposed infants of age only 6 months and above. This would introduce recall bias and selection bias respectively.

## Conclusion

Above one third of HIV positive mothers stopped breastfeeding before 12 months. The estimated KM proportions of women who were breastfeeding at 6, 9, 12 and 17 months were 89.3%, 75.3%, 66% and 17%, respectively. Monthly income, bottle feeding and family size were the independent predictors of breastfeeding cessations. In this study having low monthly income and having larger family size were associated with decreased risk of cessation while bottle feeding was associated with increased risk of breastfeeding cessation. Therefore, strengthening current counseling modality on avoidance of bottle feeding and continued breastfeeding for improved HIV free survival and further research are recommended.
